# Determination of Dipicolinic Acid through the Antenna Effect of Eu(III) Coordination Polymer

**DOI:** 10.3390/molecules29174259

**Published:** 2024-09-08

**Authors:** Jing Li, Yu Liang, Chun Tian, Hongyan Zou, Lei Zhan, Lijuan Wang, Chengzhi Huang, Chunmei Li

**Affiliations:** 1 Department of Basic Medicine, Shangqiu Medical College, Shangqiu 476100, China; liching1028@163.com; 2 Key Laboratory of Biomedical Analytics (Southwest University), Chongqing Science and Technology Bureau, College of Pharmaceutical Sciences, Southwest University, Chongqing 400715, China; 13637866124@163.com (Y.L.); tianchun2001@email.swu.edu.cn (C.T.); zhy2013@swu.edu.cn (H.Z.); zhanlei6@swu.edu.cn (L.Z.); chengzhi@swu.edu.cn (C.H.); 3 Environment and Quality Test Department, Chongqing Chemical Industry Vocational College, Chongqing 401228, China

**Keywords:** Eu(III)-coordinated polymers (Eu-CPs), antenna effect, dipicolinic acid (DPA), biomarkers of *Bacillus anthracis* spores

## Abstract

*Bacillus anthracis* is a Gram-positive bacterium that can cause acute infection and anthracnose, which is a serious concern for human health. Determining *Bacillus anthracis* through its spore biomarker dipicolinic acid (DPA) is crucial, and there is a strong need for a method that is rapid, sensitive, and selective. Here, we created Eu(III)-coordination polymers (Eu-CPs) with surfaces that have abundant carboxyl and hydroxyl groups. This was achieved by using citric acid and europium nitrate hexahydrate as precursors in a straightforward one-pot hydrothermal process. These Eu-CPs were then successfully utilized for highly sensitive DPA determination. The fluorescence (FL) emission of Eu-CPs, which is typically weak due to the coordination of Eu(III) with water molecules, was significantly enhanced in the presence of DPA. This enhancement is attributed to the competitive binding between DPA’s carboxyl or hydroxyl groups and water molecules. As a result, the absorbed energy of DPA, when excited by 280 nm ultraviolet light, is transferred to Eu-CPs through an antenna effect. This leads to the emission of the characteristic red fluorescence of Eu^3+^ at 618 nm. A strong linear relationship was observed between the enhanced FL intensity and DPA concentration in the range of 0.5–80 μM. This relationship allowed for a limit of detection (LOD) of 15.23 nM. Furthermore, the Eu-CPs we constructed can effectively monitor the release of DPA from Bacillus subtilis spores, thereby further demonstrating the potential significance of this strategy in the monitoring and management of anthrax risk. This highlights the novelty of this approach in practical applications, provides a valuable determination technique for Bacillus anthracis, and offers insights into the development cycle of microorganisms.

## 1. Introduction

*Bacillus anthracis* is a Gram-positive bacterium responsible for causing anthrax [1]. Anthrax primarily affects herbivores and is typically the result of natural infection. Humans usually become infected with anthrax through contact with infected animals or contaminated animal products [2]. Due to the high pathogenicity, lethality, and infectivity of Bacillus anthracis, the potential use of anthrax spores as a biological weapon by bioterrorists is a significant concern [3]. Consequently, there is a pressing need for accurate, rapid, and sensitive methods for the detection of Bacillus anthracis. This bacterium exhibits remarkable resilience; when environmental conditions are unfavorable for growth—such as exposure to high temperatures, high pressure, or ultraviolet light—it can enter a dormant state and produce spores [4]. When the growth environment is optimal, the spores germinate and release a variety of substances. Plasmids, such as pXO1 and pXO2, are frequently used as *Bacillus anthracis* nucleic acid determination targets. Some specific chromosomal sequences, such as *BA813*, *RpoB*, and *Gyra*, have been identified as biomarkers thanks to advances in genomics. For example, anthrax toxin is made up of three components: protective antigen (PA) [5], lethal factor (LF) [6], and edema factor (EF) [7]. PA and LF combine to form a lethal toxin, while a combination of PA and EF produces edema toxin. These toxin components are critical for determining *Bacillus anthracis* and diagnosing anthracnose. For instance, 2,6-dipicolinic acid (DPA), the main component of spores, contributes significantly to spore resistance and can be used as a *Bacillus anthracis* biomarker [8,9]. Although *BA813*, *RpoB*, *Gyra*, or PA, LF, EF, and other biomarkers can be chosen for their high accuracy and sensitivity in determining anthrax spores, the sample pretreatment and extraction processes are time-consuming and labor-intensive, require expensive equipment, etc., and result in a relatively high cost and difficult operation [10]. Therefore, we have selected DPA as an anthrax spore biomarker. In the field of DPA analysis, the primary clinical strategy for detecting anthrax involves evaluating genetic sequences and employing polymerase chain reaction (PCR) and enzyme-linked immunosorbent assay (ELISA) techniques. PCR amplifies specific DNA sequences associated with diphtheria toxin production, while ELISA utilizes specific antibodies to detect and quantify diphtheria toxin levels [10,11]. Unfortunately, the prolonged detection process for anthrax spores not only delays the rapid identification of the disease but also heightens the risk of infection. The fluorescence spectroscopy is used to detect the target quickly, sensitively, and accurately without the need for complicated operation methods, expensive instruments, and equipment [12,13,14,15,16,17].

Lanthanide ions (Ln^3+^) have low luminescence efficiency under UV/Vis illumination due to their small molar absorption coefficient and forbidden f-f transition [18]. However, it is classified as a Lewis acid because it has a high affinity for bonding with negatively charged, neutral oxygen or nitrogen atoms [19]. Because of these physicochemical properties, it can bind to the site of the corresponding small organic molecules, known as “antenna ligands”, that can participate in the energy transfer process, resulting in interesting photoluminescence properties [20]. This process, like large Stokes shifts and linear emission spectral bands, is known as the “antenna effect”. In addition, among other strong luminescent ions, such as Sm^3+^, Tb^3+^, and Dy^3+^, Eu^3+^ has a longer emission wavelength, making it widely used [21,22,23,24,25,26].

**Scheme 1:**
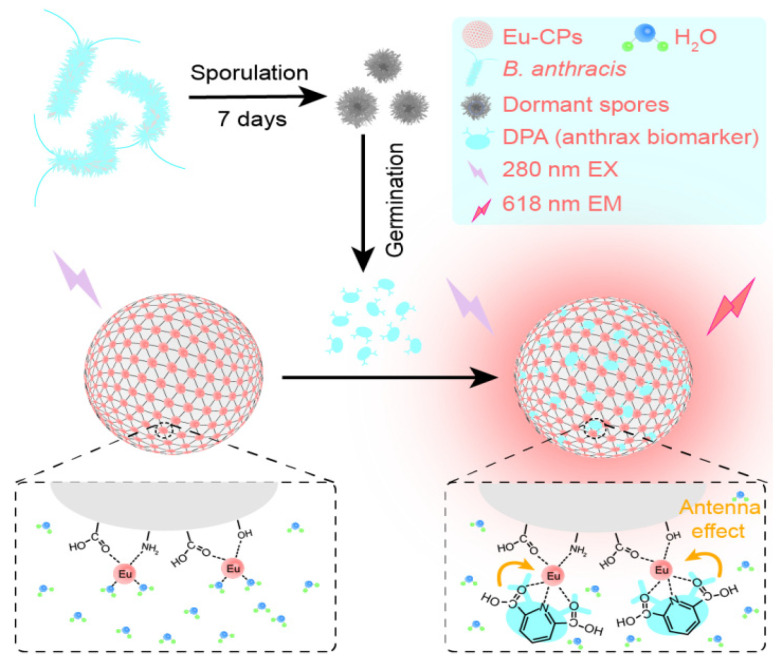
Determination illustration of DPA, one of the biomarkers of *Bacillus anthracis* spores, through the “off–on” process of the antenna effect in Eu-CPs in the absence and presence of DPA. Arrows in the figure are used to indicate the sequence or flow between steps. The color change in the diagram of Bacillus anthracis represents the process of spore formation and germination.

In this work, Eu(III)-coordinated polymers (Eu-CPs) with rich surface carboxyl and hydroxyl groups were first prepared by using citric acid and europium nitrate hexahydrate as precursors through a one-pot hydrothermal route. The synthesized Eu-CPs exhibit a low background signal, which can be attributed to the selection of ligand during material synthesis. In this context, citric acid was chosen as the ligand for several reasons: first, citric acid is abundant in carboxyl groups, and its oxygen atoms can effectively coordinate with Eu^3+^ ions; second, citric acid demonstrates greater water solubility compared to nitrogen-containing ligands; and third, citric acid occupies a portion of the coordination bonds of Eu^3+^, preventing it from sensitizing Eu^3+^ to luminescence on its own. Furthermore, the remaining coordination sites of the Eu-CPs will be occupied by water molecules from the surrounding medium, which will further diminish the background fluorescence. Upon the introduction of the *Bacillus anthracis* spore biomarker DPA, it competes with the water molecules bound to the Eu-CPs for ligand binding, leading to the saturation of the Eu^3+^ ligand bonds. Concurrently, this competition inhibits the ability of water molecules to induce luminescence. Additionally, DPA absorbs energy from external 280 nm ultraviolet light, subsequently sensitizing the characteristic fluorescence emission of Eu^3+^ through the antenna effect, thereby facilitating the highly sensitive detection of DPA. The presence of DPA, one of the biomarkers of *Bacillus anthracis* spores, causes the Eu-CPs to have strong FL emission of Eu^3+^ owing to the antenna effect of Eu-CP DPA, achieving the goal of highly sensitive DPA determination. As a practical application, the number of *Bacillus anthracis* spores was detected, and the DPA release process of *Bacillus anthracis* spores was monitored (Scheme 1), allowing for disease control and effective prevention of bacterial spore germination.

## 2. Results and Discussion

### 2.1. Characteristic Features of Eu-CPs

**Figure 1:**
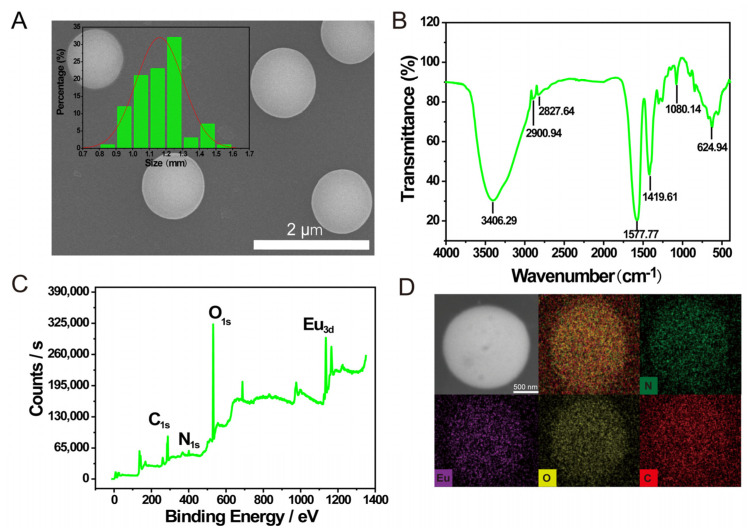
Characterization of Eu-CPs. (**A**) SEM image (inset: particle size distribution). (**B**) FT-IR spectrum. (**C**) XPS spectrum. (**D**) EDS mapping.

As shown in Figure 1A, the as-prepared Eu-CPs displayed a typical spherical structure with an average particle size of 1.16 ± 0.14 μm, as determined by the scanning electron microscopic (SEM) image, which was consistent with the hydrodynamic diameter of 1.22 ± 0.0032 μm measured by dynamic light scattering (DLS), further confirming the consistency and uniformity of the Eu-CPs. Based on the analysis of the infrared spectrum, it can be observed that the *ν*_C–H_ band [27] is associated with the absorption bands at 2900.94 and 2827.64 cm^−1^. Additionally, the *ν*_O–H,_ *ν*_C=O_, *ν*_C–N_, and *ν*_C–O_ bands are denoted by the absorption bands at 3406.29, 1577.77, 1419.61, and 1080.14 cm^−1^, respectively [28]. Furthermore, the formation of the coordination bond between Eu and –COOH (or –OH) was confirmed by the infrared absorption band at 493.78 cm^−1^ (Figure 1B) [29].

**Figure 2:**
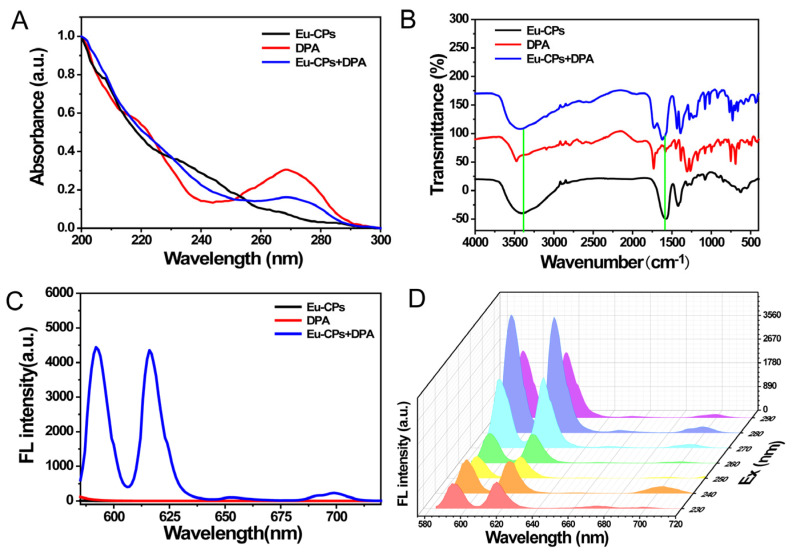
Optical properties of Eu-CPs after reacting with DPA. (**A**) UV absorption spectra of Eu-CPs, DPA, and Eu-CPs + DPA; (**B**) FT-IR spectra of Eu-CPs, DPA, and Eu-CPs + DPA. The green line marks the shift of infrared absorption peak; (**C**) fluorescence spectra of Eu-CPs, DPA, and Eu-CPs + DPA; (**D**) FL emission spectra of Eu-CPs + DPA under different excitation wavelengths from 230 to 290 nm. The line colors of the spectrum in the figure represent different excitation wavelengths.

In addition, XPS spectra confirmed that the surface functional groups of Eu-CPs correspond to the infrared spectrum results. The three prominent peaks observed at 288.6, 531.62, and 1135.58 eV were identified as C1s, O1s, and Eu3d, respectively (Figure 2C). The weaker peak at 401.58 eV was attributed to N1S. Three peaks at 284.8, 286.4, and 288.6 eV were identified in the high-resolution XPS spectra of C1s; these peaks correspond to C−C/C=C, C−O, and C=O, respectively (Figure S2A) [30]. C=O and C−OH were identified as the two peaks at 531.4 and 532.2 eV in the O1s spectrum, respectively (Figure S2B) [27]. Two peaks at 399.4 and 401.6 eV in the N1S spectrum indicated the presence of minute quantities of C−N and N−H, respectively (Figure S2C) [31]. Two peaks at 1125.5 and 1135.6 eV, which correspond to Eu(II)3d^5/2^ and Eu(III)3d^5/2^ [32], respectively, were observed in the Eu3d spectrum (Figure S2D).

The above results clearly demonstrated that the surface of Eu-CPs was covered in –COOH, –OH, and –NH2 groups. Eu^3+^ was evenly distributed on the surface of Eu-CPs, as revealed by an energy-dispersive X-ray spectroscopy (EDS) mapping image (Figure 1D).

### 2.2. Investigation of the Reaction of Eu-CPs and DPA

Figure 2A illustrates that the synthesized Eu-CPs exhibit a weak absorption band due to their small molar absorption coefficient and forbidden f-f transition. Consequently, Eu-CPs demonstrate a significantly low luminous efficiency when exposed to ultraviolet light. By incorporating DPA as an antenna ligand [33], the absorption band of DPA in the 260–280 nm range decreased, indicating the formation of the Eu-CPs-DPA structure. This structure is capable of absorbing ultraviolet light energy and transferring it to Eu-CPs through an antenna effect. As a consequence, Eu^3+^ emits its characteristic FL.

In addition, the IR absorption bands at 3406.29 and 1577.77 cm^−1^ of Eu-CPs exhibited a red shift after the addition of DPA, indicating the formation of a Eu-CPs-DPA structure through a coordination mechanism (Figure 2B) [34]. This result was further confirmed by fluorescence spectroscopy (Figure 2C). In addition, the emission of Eu^3+^ due to the antenna effect remained consistent regardless of the excitation wavelength, which ranged from 230 nm to 290 nm. The optimal excitation and emission wavelengths were found to be 280 nm and 618 nm, respectively (Figure 2D).

To achieve accurate analytical performance, we investigated the effect of environmental conditions such as acidity, salt, and light irradiation on the reaction stability of Eu-CPs and DPA. It was discovered that Eu-CPs, DPA, and Eu-CPs-DPA emit weak FL in acidic mediums because both carboxyl groups on the surface of Eu-CPs and DPA are protonated, and the coordination effect of DPA with Eu(III) is reduced, leading to reduced antenna effects and red FL [35]. The optimal pH condition was found to be 9.03 (Figure S3A). Exposing Eu-CPs to different concentrations of NaCl solution (40–200 μM) or an ultraviolet lamp for 7000 s had no significant effect on the fluorescence emission intensity with DPA (Figure S3B,C). The results showed that the newly formed Eu-CPs-DPA structure remained very stable despite changes in environmental conditions.

### 2.3. Analytical Performance of DPA

**Figure 3:**
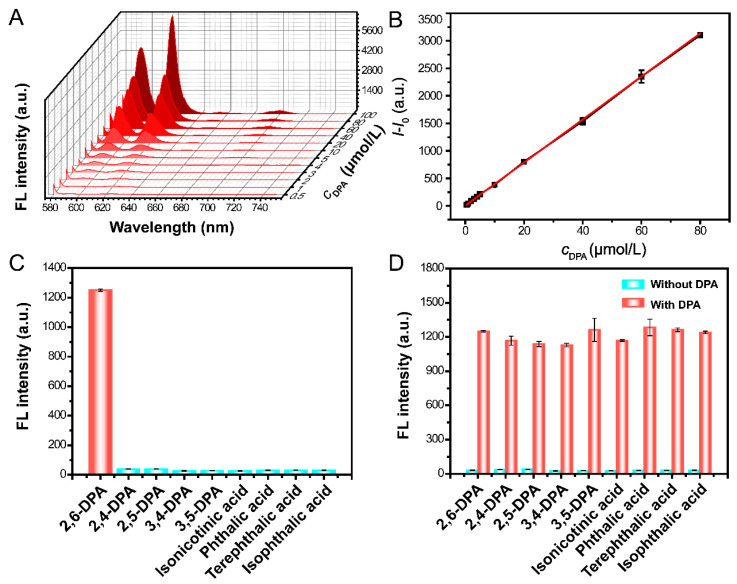
Fluorescence response, selectivity, and anti-interference of Eu-CPs for DPA determination. (**A**) The fluorescence intensity change of Eu-CPs with increasing DPA concentration. (**B**) Linear relationship between *I − I*_0_ and various DPA concentrations. (**C**) FL responses of Eu-CPs in the presence of 30 μM DPA or 200 μM of other interfering substances. Error bars represent standard deviations of three repetitive experiments (*n* = 3). (**D**) FL responses of Eu-CPs in the coexistence of 30 μM DPA and 200 μM of other interfering substances. Error bars represent standard deviations of three repetitive experiments (*n* = 3).

To investigate the analytical performance of the “fluorescence on” strategy for DPA determination under these ideal conditions, the fluorescence spectra of Eu-CPs with increasing DPA concentrations were measured. Figure 3A,B shows that FL intensity at 618 nm increased linearly with DPA concentration in the range of 0.5–80 μM, following an equation of *I − I*_0_ = 39.08*c*_DPA_ + 5.03 with a correlation coefficient (*R^2^*) of 0.999. The LOD for DPA was 15.23 nM (3*σ/k*) and the binding constant was 3.32 × 10^3^ M^−1^ (Figure S4), which is consistent with previously reported methods of DPA determination (Table S1).

Furthermore, this method has excellent selectivity and anti-interference capabilities. Figure 3C shows that only DPA causes an obvious increase in the FL emission of Eu-CPs in the presence of substances similar in structure to DPA, such as 2,4-dipicolinic acid (2,4-DPA), 2,5-dipicolinic acid (2,5-DPA), 3,4-dipicolinic acid (3,4-DPA), 3,5-dipicolinic acid (3,5-DPA), isonicotinic acid, phthalic acid, terephthalic acid, and isophthalic acid, even at concentrations nearly 7 times that of DPA. That is, Eu-CPs are highly selective and specific for DPA in aqueous solution. It should be noted that the presence of DPA in conjunction with other similar structural substances has no significant effect on the FL emission caused by the antenna effect (Figure 3D), demonstrating the excellent selectivity and anti-interference ability of the proposed method for DPA determination. We speculate that this selectivity is due to the unique molecular structure and electronic characteristics of DPA, which enable DPA to form a more stable coordination complex with Eu-CPs. Specifically, the spatial position of the two carboxyl groups of DPA may be more conducive to the effective coordination with the active sites on the surface of Eu-CPs [11,36].

### 2.4. Determination of the Bacterial Spores Amount

*Bacillus anthracis* is dormant and produces spores that initiate a self-protecting mechanism when exposed to high temperatures, high pressure, ultraviolet rays, etc. When the spores are in an environment favorable to their growth, they germinate and become pathogenic bacteria. Therefore, the quantitative evaluation of bacterial spores is extremely important [37]. For our experiments, we used *Bacillus subtilis* rather than the pathogenic *Bacillus anthracis*. To conduct spectral analysis, *Bacillus subtilis* suspension was diluted to various concentrations (1.4 × 10^8^, 2.8 × 10^8^, 4.2 × 10^8^, 5.6 × 10^8^, and 8.2 × 10^8^ spores mL^−1^) and lysed at 90 °C in dodecylamine for 1 h.

**Figure 4:**
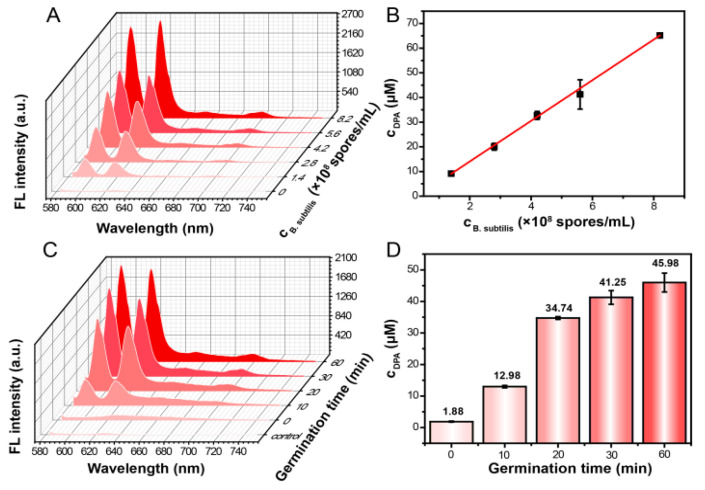
Study of biomarkers of *Bacillus anthracis* spores in actual samples by using Eu-CPs. (**A**) Fluorescence titration of Eu-CPs with different concentrations of *Bacillus subtilis* spore suspensions (1.4 × 10^8^, 2.8 × 10^8^, 4.2 × 10^8^, 5.6 × 10^8^, and 8.2 × 10^8^ spores mL^−1^) at pH 9.03 (*λ*_ex_ = 280 nm, germination time = 2 h). (**B**) Linear relationship between *c*_DPA_ and *c_Bacillus subtilis_*_spores_. (**C**) Fluorescence titration of Eu-CPs with different germination times (0, 10, 20, 30, and 60 min) of *Bacillus subtilis* suspensions at pH 9.03 (*λ*_ex_ = 280 nm; *c_Bacillus subtilis_* _spores_ = 6.18 × 10^8^ spores mL^−1^). (**D**) Released amounts of DPA from *Bacillus subtilis* spores at different germination times. Error bars represent standard deviations of three repetitive experiments (*n* = 3).

A modification in the emission signal at 618 nm was observed in each instance subsequent to the Eu-CPs treatment of the diluted suspensions. The emission intensity at 618 nm exhibits a progressive increase with the concentration of bacterial spores, as depicted in Figure 4A. A strong linear correlation was established between *I* − *I*_0_ and the spore concentration (*I − I*_0_ = 322.10, *c_Bacillus subtilis_* _spores_ = −90.16, *R*^2^ = 0.999) within the concentration range of 1.4 × 10^8^–8.2 × 10^8^ spores mL^−1^ (Table S2, Figure S5A). The obtained LOD was calculated to be 2.99 × 10^7^ spores mL^−1^. Due to the fact that every *Bacillus subtilis* spore contains 3.65 × 10^–16^ mols of DPA^25^, we were able to accurately determine the DPA concentration of various spore concentrations with relative errors ranging from −8.51% to 9.13% (Table S2; Figure 4B). This suggests that the proposed method has the potential to be utilized for the quantitative evaluation of bacterial spores.

### 2.5. DPA Monitoring of the Germination of Bacterial Spores

To further demonstrate the practical applications of this method in actual biological scenarios, the researchers investigated the variation of DPA concentration released from *Bacillus subtilis* spores within 60 min using Eu-CPs. As shown in Figure 4C,D, as the germination time increased, the amount of DPA released from *Bacillus subtilis* spores increased gradually. Bacterial germination can be divided into three phases based on DPA release behavior. Figure 4D demonstrates that the first 20 min of rapid germination resulted in a DPA concentration of 34.74 μM. *Bacillus subtilis* can grow quickly under these conditions. Therefore, *Bacillus subtilis* releases the majority of the DPA, which aids in spore germination. After 20 to 30 min, germination slowed, and DPA concentration reached 41.25 μM. The release rate of DPA was slower in the final 30 to 60 min because most of the spores had completely germinated. The measured DPA concentration was 45.98 μM, which was within 10% of the theoretical value of 45.11 μM (calculated by releasing 6.18 × 10^8^ spores mL^−1^) (Figure S5B, Table S3). These observations provide useful information about the development cycles of microorganisms.

## 3. Experimental Section

### 3.1. Materials and Reagents

All chemicals used were at least analytical reagent grade and did not require any further purification. Europium nitrate hexahydrate (Eu(NO_3_)_3_·6H_2_O) was purchased from Aladdin Chemistry Co., Ltd. in Shanghai, China. 2,6-Dipicolinic acid (DPA) and citric acid (CA) were commercially available from Merck Life Science Co., Ltd. (Shanghai, China); dodecylamine was purchased from Tokyo Chemical Industry Co., Ltd. (Tokyo, Japan); and Britton–Robinson (BR) buffer was used to control acidity. The experiment utilized ultrapure water (18.2 MΩ·cm, Mili-Q, Merck KgaA, Darmstadt, Germany).

### 3.2. Apparatus

The fluorescence (FL) spectra were obtained using an F-2500 spectrophotometer (Hitachi, Tokyo, Japan), with 5 nm slits for both excitation and emission and a photomultiplier tube (PMT) voltage of 700 V. The UV absorption spectra were measured using a Hitachi U-3010 spectrophotometer (Hitachi, Tokyo, Japan). An S-4800 SEM (Hitachi, Tokyo, Japan) was used to perform scanning electron microscopic characterizations. The infrared spectra were measured with an IR Prestige-21 instrument (Shimadzu, Tokyo, Japan). The elemental compositions were measured using an ESCALAB 250 X-ray photoelectron spectrometer (Thermo Fisher Scientific, Waltham, MA, USA). The FL lifetimes were measured using an FL-TCSPC FL spectrophotometer (Horiba Jobin Yvon, Stow, MA, USA). Hydrodynamic diameters were determined by Zetasizer Nano ZS (Malvern Instruments Ltd., Malvern, UK).

### 3.3. Synthesis of Eu-CPs

Eu-CPs were synthesized according to the procedure described in the literature [38]. Citric acid (874 mM) and Eu (NO_3_)_3_ (200 mM) were synthesized using a hydrothermal method at 160 °C for 7 h in a Teflon-lined stainless steel autoclave. The reaction product was then allowed to cool naturally before being dialyzed for 72 h with a cellulose ester dialysis membrane (500 MWCO) to remove any residual raw material. Following dialysis, a white precipitate separated. By freeze-drying, a white powdery product of Eu-CPs was produced. In total, 5 mg of Eu-CPs was dispersed in 1 mL of ultrapure water. The mixture was sonicated for 2 min and then centrifuged at 8000 rpm for 5 min to obtain a supernatant containing 0.84 mg mL^−1^ of the solution, which could be used for further processing.

### 3.4. FL Response of Eu-CPs to DPA

In a 1.5 mL tube, 200 μL of Eu-CP supernatant (0.84 mg mL^−1^) and 400 μL of BR buffer (pH 9.03) were added, followed by 200 μL of DPA (concentrations ranging from 2.5 to 500 μM). After thorough mixing, the solution was diluted to 1.0 mL with doubly distilled water and blended again. FL spectra were recorded immediately after mixing in the 560–700 nm range, with excitation at 280 nm.

### 3.5. Bacterial Spore Culture

Because both Bacillus anthracis and *Bacillus subtilis* have the same spore biomarker, dipicolinic acid (DPA), we chose *Bacillus subtilis* over the infectious Bacillus anthracis for the sake of safety. Bacterial cultivation was carried out using the previously described method [39]. For 7 days, third-generation *Bacillus subtilis* was cultured in LB liquid medium (10 mg mL^−1^ tryptone, 5.0 mg mL^−1^ yeast extract, 10 mg mL^−1^ NaCl) at 37 °C and 200 rpm. After cultivation, the bacterial spore suspension was centrifuged at 6000 rpm for 10 min, washed 5 times with sterile water, diluted to 3.75 × 10^9^ spores mL^−1^, and stored at 4 °C in a refrigerator for future use.

### 3.6. DPA Determination during Spore Germination

To germinate and release DPA, a fresh suspension of *Bacillus subtilis* spores (1.4 × 10^8^ to 8.2 × 10^8^ spores mL^−1^) was diluted with sterile water and heated to 90 °C for 1 h with 0.66 mM of dodecylamine. Following that, the activated spore suspension was centrifuged at 10,000 rpm for 3 min to obtain the supernatant. Then, 100 μL of the supernatant was added to a mixture containing 200 μL of Eu-CPs (0.84 mg mL^−1^) and 400 μL of BR buffer (pH 9.03), and the mixture was diluted to a final volume of 1 mL with ultrapure water. Immediately after mixing, the emission spectrum of the sample was recorded.

To activate the spores and release DPA, a fresh suspension of *Bacillus subtilis* was diluted to 6.18 × 10^8^ spores mL^−1^ with sterile water and heated to 90 °C for different time intervals (0, 10, 20, 30, and 60 min) with 0.66 mM of dodecylamine. The FL spectra were measured using the same procedure as described above.

## 4. Conclusions

In this study, we successfully synthesized Eu(III)-coordination polymers (Eu-CPs) and utilized them for the highly sensitive detection of DPA (2,6-dipyridine acid). Due to their abundant surface carboxyl and hydroxyl groups, Eu-CPs significantly enhance fluorescence emission in the presence of DPA through the antenna effect. We observed that the competitive binding between DPA and Eu-CPs leads to substantial energy transfer, thereby triggering the characteristic red fluorescence emission of Eu^3+^. Using this method, we achieved a linear detection of DPA concentrations ranging from 0.5 to 80 μM, with a detection limit as low as 15.23 nM and a binding constant of 3.32 × 10^3^ M^−1^. Furthermore, our study demonstrates the potential applications of Eu-CPs in real biological contexts, including the quantitative assessment of bacterial spore numbers and the monitoring of DPA release during spore germination. We validated the accuracy of this method for detecting spore numbers by comparing the results with theoretical values. With this method, we observed that the amount of DPA released from *B. subtilis* spores increased gradually with longer germination times, providing valuable insights for the study of the developmental cycles of microorganisms. Our results emphasize the potential of Eu-CPs to detect DPA, the biomarker of Bacillus anthracis, which provides a scientific basis for developing new biological threat detection and prevention strategies. The probe is expected to provide valuable information for studying the growth cycle of microorganisms and preventing the development of harmful pathogens in the future.

## Data Availability

Data are contained within the article and Supplementary Materials.

## Supplementary Materials

The following supporting information can be downloaded at: https://www.mdpi.com/article/10.3390/molecules29174259/s1, Figure S1: Dynamic light scattering characterization of Eu-CPs; Figure S2: Characterization of Eu-CPs; Figure S3: The stability experiments of the Eu-CPs + DPA; Figure S4: Benesi-Hildebrand plot of Eu-CPs with DPA; Figure S5: The linear relationship between *I − I*_0_ and c*_Bacillus subtilis_*_spores_. The concentration of *Bacillus subtilis* activation corresponding to different germination time; Table S1: Comparison of DPA determination between this work and other strategies; Table S2: Different concentrations of *Bacillus subtilis* spores are completely released to obtain the theoretical and actual values of different concentrations of DPA; Table S3: Concentrations of DPA released by *Bacillus subtilis* spores at different germination times. Refs. [40,41,42,43,44,45] are cited in the Supplementary Materials.
