# Supplementary material for: Determination of Dipicolinic Acid through the Antenna Effect of Eu(III) Coordination Polymer
Source: Molecules. 2024 Sep 8;29(17):4259. doi: 10.3390/molecules29174259 (PMC11397212; doi:10.3390/molecules29174259)
Supplement: Supplementary file 1 [file molecules-29-04259-s001.zip › molecules-3158721-supplementary.pdf]

Supporting Information (SI)

Determination of Dipicolinic Acid through the Antenna Effect of Eu(III) Coordination Polymer

Jing Li † [1,2], Yu Liang † [2], Chun Tian [2], Hongyan Zou [2], Lei Zhan [2], Lijuan Wang [1,*], Chengzhi Huang [2], Chunmei Li [2,*]

[1] Department of Basic Medicine, Shangqiu Medical College, 476100.

[2] Key Laboratory of Biomedical Analytics (Southwest University), Chongqing Science and Technology Bureau, College of Pharmaceutical Sciences, Southwest University, Chongqing 400715, P. R. China.

[*] Corresponding authors. E-mail: wlj.wang@163.com, licm1024@swu.edu.cn

[†] These authors contributed equally to this work.

Supplementary Figures

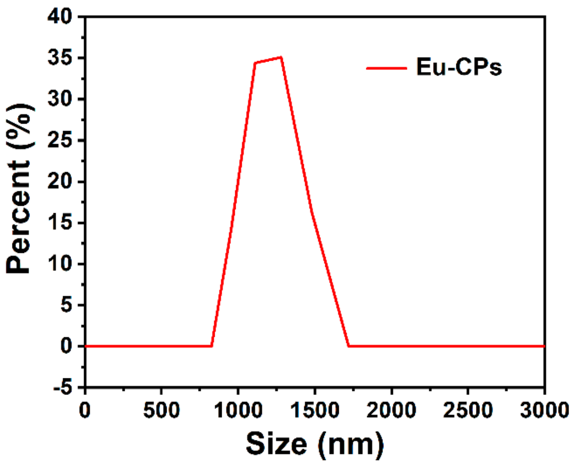

Figure S1. Dynamic light scattering characterization of Eu-CPs.

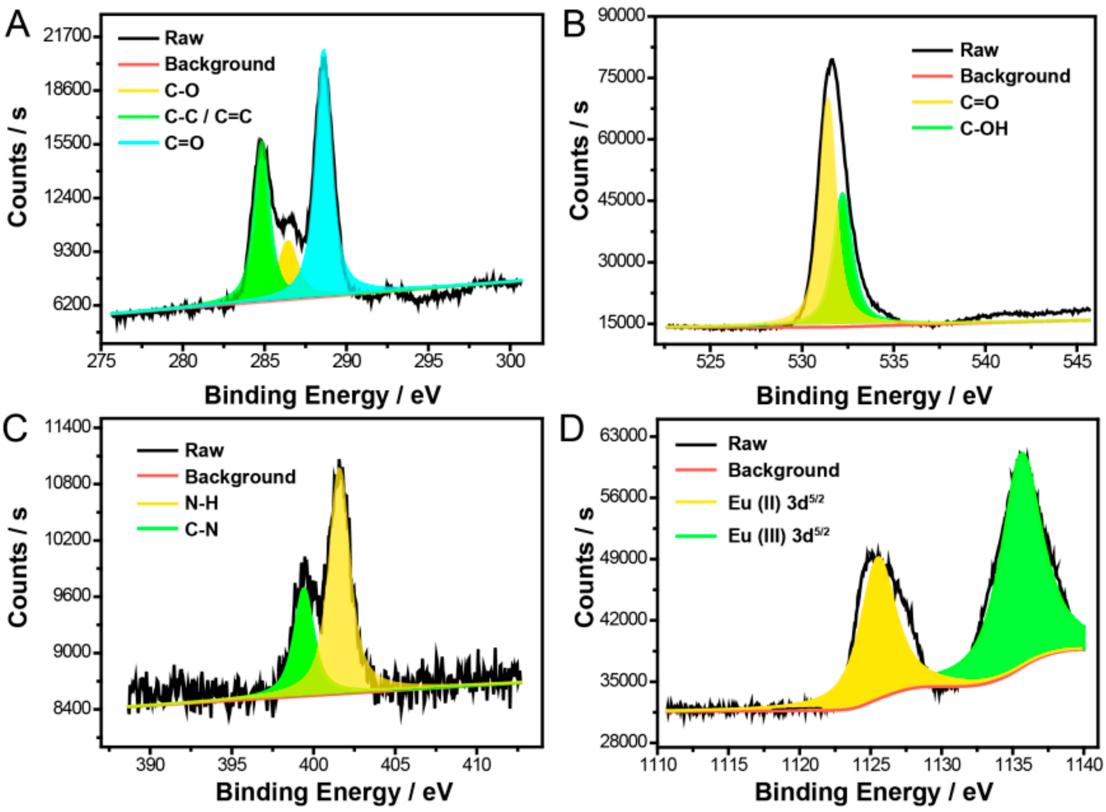

Figure S2. Characterization of Eu-CPs. High-resolution XPS spectra of (A) C_{1s}, (B) O_{1s}, (C) N_{1s}, and (D) Eu_{3d}.

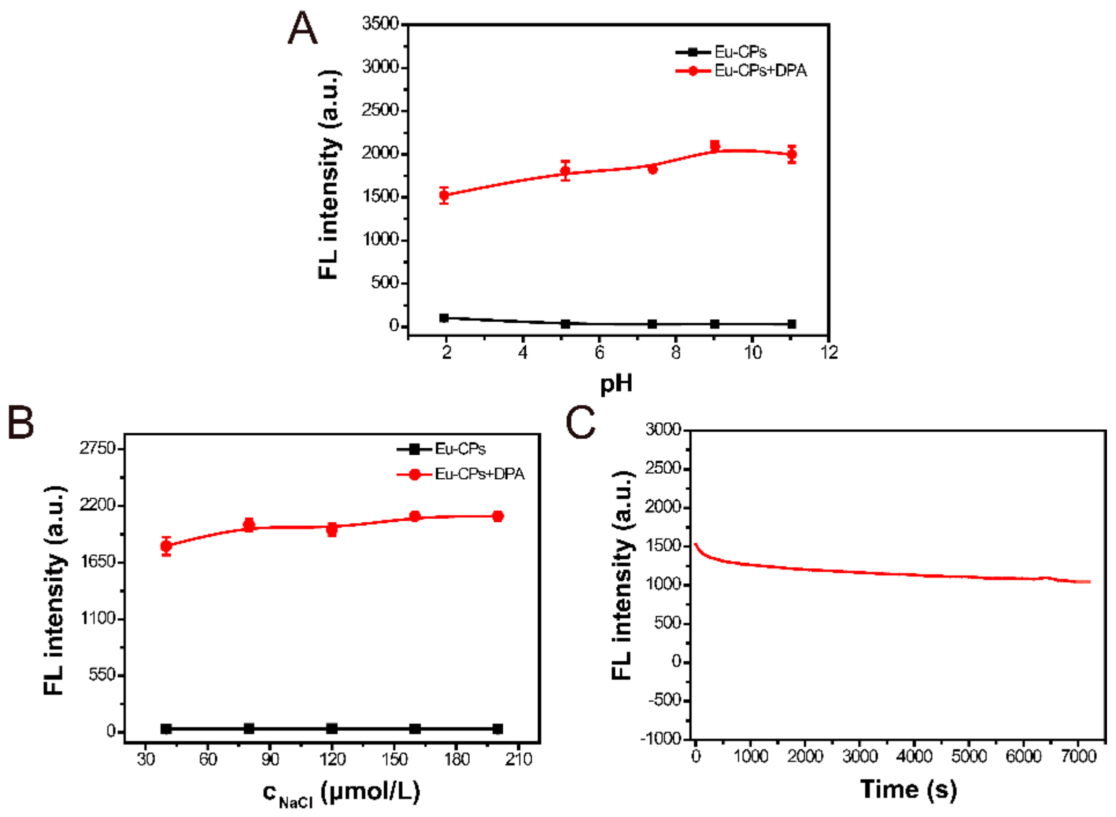

Figure S3. The stability experiments of the Eu-CPs+DPA (A) The influence of pH environment on the fluorescence intensity of Eu-CPs to recognize DPA. (B) Salty stability (NaCl, 40, 80, 120, 160 and 200 mM). (C) The ability of anti-photobleaching. Error bars represent standard deviations of three repetitive experiments (n = 3).

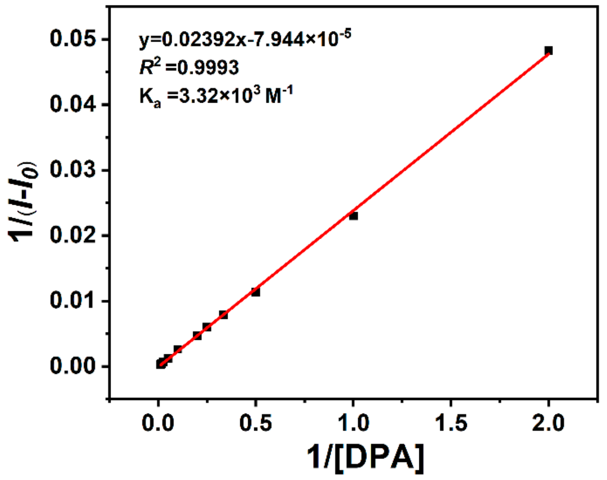

Figure S4. Benesi-Hildebrand plot of Eu-CPs with DPA

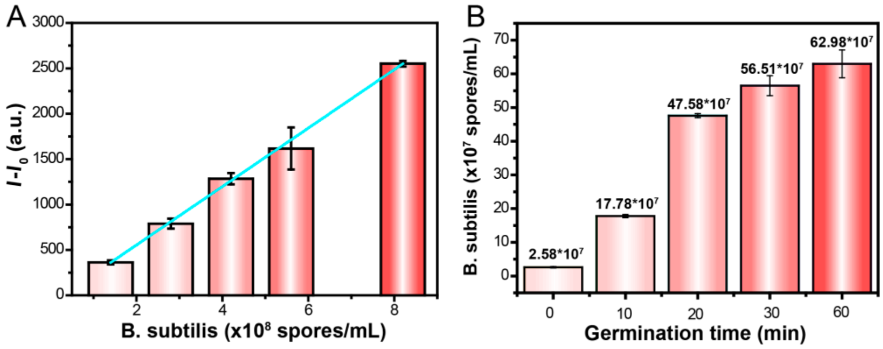

Figure S5. (A) the linear relationship between I-I_0 and $c_{Bacillus\ subtilis\ \mathrm{spores}}$ (1.4×10^8, 2.8×10^8, 4.2×10^8, 5.6×10^8 and 8.2×10^8 spores mL^{-1}, λ_{ex}=280 nm, germination time=2 h). (B) The concentration of *Bacillus subtilis* activation corresponding to different germination time (λ_{ex}=280 nm; $c_{Bacillus\ subtilis\ \mathrm{spores}}$=$6.18\times10^8$ spores mL^{-1}). Error bars represent standard deviations of three repetitive experiments (n = 3).

Supplementary Tables

Table S1. Comparison of DPA determination between this work and other strategies.

Probe	Linear range (μM)	LOD (nM)	Ref.
Sm^{3+}/Au(0)@Au(I)−SG NC	1-120	100.0	[1]
TbP-CPs	0-8	5.0	[2]
BCNO QD−EDTA−Eu^{3+}	0-2.5	0.5	[3]
XO-CdTe QDs	0.1-5	42.0	[4]
Eu@SiNPs	0.5-20	150.0	[5]
$Tb_{0.875}Eu_{0.125}$-Hddb	0-100	849.0	[6]
INT-probes	0.1-7	100.0	[7]
Eu/Tb-MOFs	0-600	248.0	[8]
Tb0.4/Eu0.6-BTC	0-3	4.9	[9]
Tb-PTA-OH MOF	0-6	13.4	[10]
Eu-CPs	0.5-80	15.2	This work

Table S2. Different concentrations of *Bacillus subtilis* spores are completely released to obtain the theoretical and actual values of different concentrations of DPA.

Spores mL^{-1} (*10^8)	DPA conc. theoretical (μM)	DPA conc. actual (μM)	RSD (*n*=3, %)	Relative error (%)[a]
1.4	10	9.15	6.31	-8.51
2.8	20	20.06	6.99	0.29
4.2	30	32.74	4.92	9.13
5.6	40	41.24	14.40	3.11
8.2	60	65.16	1.227	8.60

a: Relative error (%) = (DPA conc. actual-DPA conc. theoretical)/DPA conc. theoretical*100. 1 spore mL^{-1} of *Bacillus subtilis* ~ 3.65×10^{-16} moles of DPA. 0.2 M BR buffer (pH 9.03). λex= 270 nm. λem=618 nm.

Table S3. Concentrations of DPA released by *Bacillus subtilis* spores at different germination times.

Germination time (min)	DPA conc. actual (μM)			RSD (*n*=3, %)
0	1.70	1.95	2.00	8.41
10	12.77	12.79	13.40	2.78
20	35.17	34.74	34.30	1.25
30	41.85	43.06	38.85	5.25
60	43.84	44.67	49.42	6.55

Conc. of stock *Bacillus subtilis* suspension=6.18×10^8 spores mL^{-1} (Theoretical value of DPA was 45.11 μM). 1 spore mL^{-1} of *Bacillus subtilis* ~ 3.65×10^{-16} moles of DPA. 0.2 M BR buffer (pH 9.03). λex=270 nm; λem=618 nm.

References

1. Halawa, M. I.; Li, B. S.; Xu, G. B., Novel synthesis of thiolated gold nanoclusters induced by lanthanides for ultrasensitive and luminescent detection of the potential anthrax spores' biomarker. *ACS Appl. Mater. Interfaces* **2020,** *12* (29), 32888-32897.
2. Luo, Y. Q.; Zhang, L.; Zhang, L. Y.; Yu, B. H.; Wang, Y. J.; Zhang, W. B., Multiporous terbium phosphonate coordination polymer microspheres as fluorescent probes for trace anthrax biomarker detection. *ACS Appl. Mater. Interfaces* **2019,** *11* (17), 15998-16005.
3. Rong, M. C.; Yang, X. H.; Huang, L. Z.; Chi, S. T.; Zhou, Y. B.; Shen, Y. E.; Chen, B. Y.; Deng, X. Z.; Liu, Z. Q., Hydrogen peroxide-assisted ultrasonic synthesis of BCNO QDs for anthrax biomarker detection. *ACS Appl. Mater. Interfaces* **2019,** *11* (2), 2336-2343.
4. Cao, Y. T.; Gong, X. L.; Li, L.; Li, H. H.; Zhang, X. M.; Guo, D. Y.; Wang, F. X.; Pan, Q. H., Xylenol orange-modified CdTe quantum dots as a fluorescent/colorimetric dual-modal probe for anthrax biomarker based on competitive coordination. *Talanta* **2023,** *261*, 124664.
5. Na, M.; Zhang, S. P.; Liu, J. J.; Ma, S. D.; Han, Y. X.; Wang, Y.; He, Y. X.; Chen, H. l.; Chen, X. G., Determination of pathogenic bacteria—Bacillus anthrax spores in environmental samples by ratiometric fluorescence and test paper based on dual-emission fluorescent silicon nanoparticles. *J. Hazard. Mater.* **2020,** *386*, 121956.
6. Chen, X. B.; Qi, C.-X.; Xu, Y.-B.; Li, H.; Xu, L.; Liu, B., A quantitative ratiometric fluorescent Hddb-based MOF sensor and its on-site detection of the anthrax biomarker 2,6-dipicolinic acid. *J. Mater. Chem. C* **2020,** *8* (48), 17325-17335.
7. Wu, J. F.; Chen, P. P.; Chen, J.; Ye, X. X.; Cao, S. R.; Sun, C. Q.; Jin, Y.; Zhang, L. Y.; Du, S. H., Integrated ratiometric fluorescence probe-based acoustofluidic platform for visual detection of anthrax biomarker. *Biosens. Bioelectron.* **2022,** *214*, 114538.
8. Shen, M. L.; Liu, B.; Xu, L.; Jiao, H., Ratiometric fluorescence detection of anthrax biomarker 2,6-dipicolinic acid using hetero MOF sensors through ligand regulation. *J. Mater. Chem. C* **2020,** *8* (13), 4392-4400.
9. Wu, M.; Jiang, Z. W.; Zhang, P.; Gong, X.; Wang, Y., Energy transfer-based ratiometric fluorescence sensing anthrax biomarkers in bimetallic lanthanide metal-organic frameworks. *Sens. Actuators, B* **2023,** *383*, 133596.
10. Yu, L.; Feng, L. X.; Xiong, L.; Li, S.; Wang, S.; Wei, Z. Y.; Xiao, Y. X., Portable visual assay of Bacillus anthracis biomarker based on ligand-functionalized dual-emission lanthanide metal-organic frameworks and smartphone-integrated mini-device. *J. Hazard. Mater.* **2022,** *434*, 128914.
